# Timing of Prostin E2 Administration After Poor Response to Propess: Impact on Induction-to-Birth Interval and Maternal/Neonatal Outcomes

**DOI:** 10.3390/medicina61071255

**Published:** 2025-07-10

**Authors:** Ning-Shiuan Ting, Yu-Chi Wei, Dah-Ching Ding

**Affiliations:** 1 Department of Obstetrics and Gynecology, Hualien Tzu Chi Hospital, Buddhist Tzu Chi Medical Foundation, Tzu Chi University, Hualien 970, Taiwan; michelleting0627@gmail.com; 2 Institute of Medical Sciences, Tzu Chi University, Hualien 970, Taiwan

**Keywords:** prostaglandin, Propess, Prostin E2, induction of labor, Bishop score

## Abstract

*Background and Objectives:* For many patients, the induction-to-delivery interval is shorter with Propess than with Prostin E2. However, some patients also require Prostin E2 to sufficiently boost their dinoprostone levels to achieve cervical change and vaginal delivery. In this study, we compared the efficacy of different timings of Prostin E2 administration after Propess use. *Materials and Methods:* This single-institution retrospective cohort study was conducted between January 2020 and August 2023. The inclusion criterion was an unfavorable cervix (Bishop score ≤ 6) after Propess use for 8 h. We divided the patients into three groups based on the addition of Prostin E2 at the 8th (group 1), 12th (group 2), and 24th (group 3) hour after Propess insertion. The primary outcome was the cesarean section rate. The secondary outcomes were the induction-to-birth interval, Bishop score at 24 h, neonatal outcomes, and the predictors of labor induction duration. *Results:* A total of 63 patients were analyzed across three groups based on the timing of Prostin E2 administration (8, 12, and 24 h). The gestational age differed significantly between groups (*p* < 0.001), with the highest age being observed in the 24 h group. The 8 h group had the shortest induction-to-birth interval (*p* < 0.001) and the highest Bishop scores after 24 h of Propess use (*p* < 0.001). Blood loss was lowest in the 12 h group (*p* = 0.027). No significant differences were found in relation to the mode of birth, tachysystole, neonatal birth weight, or Apgar scores. A multivariable analysis identified gestational age (β = 3.33; *p* = 0.015) and Bishop score after 24 h of PGE2 (β = −1.99; *p* < 0.001) as being independent predictors of labor duration. *Conclusions:* administering Prostin E2 to patients who had a poor response after Propess use was safe; additionally, adding it at the 8th hour after Propess initiation could result in a shorter induction-to-birth interval.

## 1. Introduction

Induction of labor (IOL), defined as the stimulation of contractions before the spontaneous onset of labor, has been performed at an increasing rate in recent decades [1]. IOL is a common obstetric procedure, with prevalence rates varying across studies, ranging from 4.4% to 20.4% [2,3]. Elective induction of labor in low-risk nulliparous patients at 39 weeks of gestation is also approved, given the results of the ARRIVE randomized trial, which showed this group to have a decreased risk of cesarean section without significant differences in perinatal outcomes [4]. Successful IOL is associated with reduced stillbirths and perinatal deaths [2].

IOL is a standard procedure when prompt delivery is necessary [5], and pre-induction cervical ripening is essential for successful labor induction. The choice of method depends on various factors, including cervical assessment, safety, cost, and patient preference [5]. Prostaglandin E2 (PGE2, Prostin, 3 mg dinoprostone, Sanico N.V., Turnhout, Belgium) is commonly applied to achieve cervical ripening and IOL [4,6].

Propess (dinoprostone, Ferring, Saint-Prex, Switzerland) is a dinoprostone vaginal insert containing 10 mg of dinoprostone and has the controlled release (0.3 mg/h) characteristic of a single-dose application [7]. A single insertion has the advantage of reducing the number of vaginal examinations and can be easily removed when tachysystole or fetal distress is encountered. There was a positive linear relationship between the amount of dinoprostone released and the duration of insertion in women with intact membranes for over 24 h. However, the dinoprostone release rate is dependent on vaginal pH, with a faster release rate at a higher vaginal pH [7,8].

Comparative studies have shown no significant differences in failed induction rates between slow-release pessaries and vaginal gels for cervical ripening [9,10]. However, slow-release pessaries of PGE2 (Propess) may offer advantages, such as shorter induction-to-birth intervals and fewer vaginal examinations [10]. According to previous studies, the successful vaginal delivery rate of Propess lies between 70% and 90%, with a cesarean section rate of 10–20% owing to failure to progress. Therefore, some patients still showed a poor response after Propess use [11,12].

The optimal management for failed PGE2 induction remains unclear, with options including repeated PGE2 administration or switching to oxytocin [13]. Ongoing research aims to determine the most effective approach for achieving vaginal delivery after initial PGE2 failure, which could help establish clinical guidelines for this common obstetric scenario [13].

The efficacies of Propess and Prostin in labor induction have been compared, and studies have shown mixed results regarding delivery outcomes. One study found that Propess led to extended hospital stays and greater induction-to-delivery times compared with Prostin [14]. Another study reported slower induction and lower vaginal delivery rates compared with Propess [15]. However, a more recent study observed shorter induction-to-birth intervals and higher rates of vaginal delivery within 24 h using Propess [10]. Cesarean section rates are generally similar between the two methods [10,16]. Propess was associated with fewer vaginal examinations [10] and a reduced need for oxytocin during labor [14]. Both methods were found to be safe and effective for labor induction, with comparable complication rates [16].

The primary objective of this study was to compare the cesarean section rates among patients who received Prostin E2 at different time points (8, 12, and 24 h) following Propess insertion for labor induction. Secondary objectives included evaluating the induction-to-birth interval, assessing the Bishop score (BS) at 24 h, comparing neonatal outcomes such as birth weight and Apgar scores among the three groups, and identifying independent predictors of labor induction duration, including maternal age and cervical status.

## 2. Materials and Methods

### 2.1. Ethics Statements

This study was approved by the Research Ethics Committee of Hualien Tzu Chi Hospital (IRB number: IRB114-007-B). The requirement for informed consent was waived because of the low risk to the patients in the study, owing to the retrospective nature of the study and the use of de-identified data.

### 2.2. Study Design and Population

Our study is a retrospective analysis based on several years of clinical observation. Given that the relevant data were already available, this design allowed us to efficiently explore potential associations and trends. This single-institution retrospective cohort study was conducted between January 2020 and August 2023.

#### Inclusion and Exclusion Criteria

Inclusion criteria included nulliparous women with a singleton pregnancy at greater than 37 weeks’ gestation, cephalic presentation, and an unfavorable cervix (Bishop score ≤ 6) at 8 h after Propess insertion. A poor response to Propess was defined as a Bishop score ≤ 6 at 8 h after application.

The exclusion criteria were multiparous pregnancy, any contraindications to vaginal delivery, and rupture of membranes before labor induction. Gestational age was determined based on the Naegele rule [17].

### 2.3. Procedure of Induction of Labor

**Figure 1:**
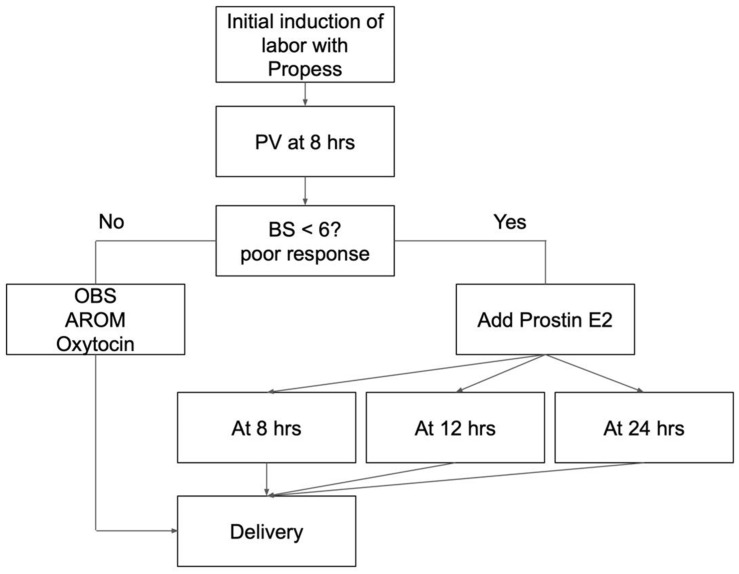
Induction of labor research protocol. PV: pelvic examination; BS: Bishop score; OBS: observation; hrs: hours; AROM: artificial rupture of membranes.

The expectant mother was admitted to the delivery room, and the fetal heart rate was monitored for 30 min. The initial vaginal examination coincided with the insertion of Propess. Subsequent examinations were scheduled based on uterine contractions and patient symptoms. Artificial rupture of membranes (AROM) was performed when the Bishop score exceeded 8. Routine vaginal examination was performed 8 h after Propess insertion. If poor progress was noted (Bishop score ≤ 6 at 8 h after Propess insertion), intravaginal Prostin E2 every 4 h was initiated either 8, 12, or 24 h after Propess insertion for augmentation if uterine contractions were insufficient (Figure 1). The procedure was concluded in the event of membrane rupture, non-reassuring fetal heart rate, or uterine tachysystole.

In our study, we defined “poor response” to Propess as BS 6 was not achieved (unfavorable cervix) after using Propess for 8 h. The cut-off value of 8 h was suggested from the result of Hung, C. H et al. which concluded that the participants with successful vaginal delivery had significantly shorter cervical length at 8 h after dinoprostone application [12]. We included the cases that met the definition of poor response after using Propess for 8 h and divided them into different intervention (Prostin E2) timings to evaluate the induction interval outcome.

The Propess is typically inserted for 12–24 h to aid cervical ripening because of its slow-release characteristic. However, clinical observations in our hospital have shown that some patients still present with an unfavorable cervix after the standard 12 to 24 h insertion period. In response, obstetricians at our institution have adopted various methods to improve outcomes, including the addition of Prostin E2 at different time points following Propess insertion. Some obstetricians choose to administer Prostin E2 after 8 h if the cervix remains unfavorable, while others prefer to wait until after 24 h. This variation in clinical practice prompted the present retrospective study, which aims to compare the efficacy of different intervention periods of Prostin E2 after failed Propess for IOL.

### 2.4. Data Collection

The basic characteristics of the patients included gestational age (GA), age, body mass index (BMI), Bishop score, labor time, and delivery mode. Neonatal outcomes, including Apgar scores and neonatal weight, were also collected.

### 2.5. Outcome Measures

We divided the patients into three groups according to the timing of the Prostin E2 intervention at the 8th (group 1), 12th (group 2), and 24th (group 3) hour after Propess insertion. The primary outcome was the cesarean section rate, and the secondary outcomes were induction-to-birth interval, Bishop score at 24 h, neonatal outcomes, and predictors of labor induction duration.

### 2.6. Statistical Analysis

Normally distributed data were expressed as mean ± standard deviation, and differences between group means were assessed using the independent-samples *t*-test. Number (%) and group differences were analyzed using the chi-square test. Multivariate regression analysis was used to predict variables associated with the outcomes. Statistical significance was set at *p* < 0.05. SPSS version 25 (IBM Corp., Armonk, NY, USA) was used for all the statistical analyses.

## 3. Results

### 3.1. Basic Characteristics of Patients

Table 1 presents the basic characteristics of the three groups. GA differed significantly among the three groups (the greatest gestational age was noted in the 24 h group; *p* < 0.001). No significant differences in age, BMI, painless labor, AROM, oxytocin use, or Bishop score at Propess insertion were observed.

### 3.2. Maternal Outcomes

Among the three groups based on the timing of Prostin E2 administration (8, 12, and 24 h), significant differences were observed in blood loss, BS at administration, BS after 24 h, and induction-to-birth interval for vaginal deliveries (Table 2). The 12 h group had the lowest blood loss (*p* = 0.027), while the 8 h group showed the highest BS after 24 h of Propess use (*p* < 0.001). The induction-to-birth interval was shortest in the 8 h group and longest in the 24 h group among vaginal births (*p* < 0.001). No significant differences were found in the rates of tachysystole, variable deceleration, mode of birth, or cesarean birth intervals. Overall, the earlier administration of Prostin E2 (particularly at 8 h) may be associated with more favorable outcomes in terms of cervical ripening and shorter labor duration.

According to the indications of C/S, five cases were related to prolonged labor with active phase arrest (one case in the 8 h group, two cases in the 12 h group, and two cases in the 24 h group) and four cases were related to prolonged labor with arrest of descent (one case in the 8 h group, one case in the 12 h group, and two cases in the 24 h group). One case was related to fetal distress in the 24 h group.

### 3.3. Neonatal Outcomes

Table 3 presents the neonatal outcomes based on the timing of Prostin E2 administration. There were no significant differences among the groups in terms of birth weight. Similarly, Apgar scores at 1 min and at 5 min were comparable across the three groups, indicating no adverse impact on immediate neonatal condition.

### 3.4. Risk Factors Associated with the Duration of Labor Induction

In the univariable analysis (Table 4), GA, blood loss, oxytocin use, and Bishop scores after 12 and 24 h of PGE2 were significantly associated with the outcome. However, in the multivariable model (Table 4), only GA (β = 3.33; *p* = 0.015) and Bishop scores after 24 h of PGE2 (β = −1.99; *p* < 0.001) remained statistically significant. Age and BMI showed borderline significance (*p* = 0.051 and *p* = 0.053, respectively), while other variables such as painless labor, AROM, oxytocin use, and Bishop scores at Propess lost significance after adjustment. This suggests that GA and the Bishop score after 24 h of PGE2 are independent predictors in the multivariable model.

## 4. Discussion

In this study, we compared three different timings of Prostin E2 administration in patients with a poor response to Propess. Our results showed that in cases of poor response to Propess use, the early administration of Prostin E2 at 8 h after Propess insertion resulted in a significantly shorter induction-to-birth interval for normal deliveries, without increasing adverse maternal or neonatal outcomes. GA and Bishop scores 24 h after Propess insertion were significant predictors of the induction-to-birth interval.

In women with an unfavorable cervix, both pharmacological and mechanical methods are effective [5]. However, few studies have evaluated the efficacy of a second dose of dinoprostone or oxytocin in patients who did not respond to the first dose of Propess. Antonazzo et al. compared the repeated vaginal insertion of Prostin and intravenous oxytocin for labor induction in patients with an unfavorable cervix that was not responsive to a first dose of Propess [18]. This study showed that with repeated vaginal application of dinoprostone, the rate of vaginal deliveries was significantly higher (55.3% vs. 34.0%; *p* < 0.05), and the rate of cesarean sections was substantially lower (44.7% vs. 66%; *p* < 0.05) [18]. However, another randomized study comparing two groups, one receiving oxytocin and the other Propess (oxytocin was delivered after 24 h of Propess use), found that the cesarean section rate was similar in the two groups (23.8% vs. 26.3%; *p* = 0.71), but the interval between treatment and delivery was significantly longer (28.1 h vs. 9.7 h; *p* < 0.0001) in the Propess group [19]. In our study, we chose Prostin E2 as the dinoprostone administered after a poor response to Propess. The cesarean delivery rate and the induction-to-birth interval in our study were higher in the group with Prostin E2 insertion at 24 h. The addition of oxytocin in patients with an unfavorable cervix causes a high failure rate, especially in nulliparous patients with a Bishop score  ≤  3. Among these patients, 66% had induction failure, and 65.4% underwent cesarean section. If the Bishop score was 4–6, the rate of induction failure decreased to 10% [19].

Beyond selecting the optimal treatment regimen for a poor response to a first dose of Propess, the timing of the intervention is also an important issue. Previous studies usually intervened 24 h after patients failed to respond to a first dose of dinoprostone [18,19]. However, in our clinical experience, the efficacy of Propess decreases over time, as the vaginal environment and room temperature affect the vaginal pessary release and absorption [7,8]. Early intervention before 24 h might be reasonable for shortening the induction-to-birth interval. In our study, when we observed a poor response to Propess after 8 h of use and performed additional interventions at 8, 12, and 24 h after Propess insertion, we found that adding Prostin E2 at 8 h resulted in a shorter induction-to-birth interval. The Bishop score at 24 h after Propess insertion, a significant predictor of the induction-to-birth interval, was also higher in the 8 h group.

Transvaginal color Doppler ultrasonography may serve as a complementary tool in guiding labor induction strategies [20]. By evaluating cervical vascularity and uterine artery blood flow, Doppler indices can reflect the physiologic readiness of the cervix and the effect of prostaglandin treatment. Intravaginal misoprostol administration has been shown to increase uteroplacental vascular resistance, though it likely does not significantly impair placental perfusion [21,22]. Therefore, in patients with a poor Bishop score but Doppler findings suggestive of cervical ripening, clinicians may consider proceeding with Prostin E2 administration. Moreover, serial Doppler assessments could offer a real-time, non-invasive method to evaluate the impact of Prostin E2 on the induction-to-birth interval.

Our study found more blood loss in the 8 h and 24 h groups compared to the 12 h group. IOL in singleton pregnancies has shown mixed effects on postpartum blood loss. While one study found no significant increase in postpartum hemorrhage (PPH) rates or estimated blood loss with IOL [23], others reported a higher incidence of PPH and increased blood loss compared to spontaneous labor onset [24,25]. IOL was associated with longer labor duration, which may contribute to increased blood loss [25]. In twin pregnancies, cesarean delivery after failed IOL was linked to higher maternal morbidity, including increased blood loss, compared to successful vaginal delivery [26]. Factors associated with successful IOL in twin pregnancies included multiparity and maternal age < 35 years [26]. Overall, the relationship between IOL and postpartum blood loss remains complex, with some studies suggesting increased risk, particularly in nulliparous women [24,25].

Regarding blood loss, both the 8 h and 24 h groups experienced significantly greater amounts of blood loss compared to the 12 h group. Although there were no significant differences in baseline characteristics among the three groups—except for gestational age—the 12 h group had a relatively lower maternal age and a lower incidence of painless labor. Previous studies have indicated that advanced maternal age (defined as >35 years) is associated with an increased risk of obstetric complications and is an independent risk factor for excessive blood loss [27,28]. While the average maternal age in our study did not meet the criteria for advanced maternal age, there may be a linear relationship between maternal age and blood loss that warrants further investigation. As for the use of painless (e.g., epidural analgesia), prior studies have shown no significant association with postpartum blood loss [29].

Tachysystole was rare in our study. Tachysystole during labor induction with prostaglandins has been a subject of research due to concerns about potential adverse outcomes. A study by Bofill et al. found that tachysystole was not associated with adverse perinatal outcomes compared to women without tachysystole during cervical ripening and labor induction [30]. However, non-reassuring fetal tracings were more common in the tachysystole group. Pierce et al. emphasized the importance of considering patient characteristics and risk factors when choosing between prostaglandins for cervical ripening [31]. Sims highlighted a legal case where prolonged tachysystole during oxytocin-induced labor raised concerns about fetal gas exchange and potential complications [32]. Kawakita et al. compared misoprostol and prostaglandin E2 for labor induction in women with oligohydramnios, finding no significant differences in cesarean delivery rates or neonatal outcomes [33]. These studies underscore the need for careful monitoring and individualized approaches when using prostaglandins for labor induction.

In our study, GA was associated with the induction-to-delivery interval. A cervical length less than 25 mm is associated with shorter intervals [34]. Maternal age, body mass index, prior vaginal delivery, gestational age, Bishop score, and induction agent significantly affect time to anesthesia and delivery [35]. Notably, delaying elective induction beyond 39 weeks of gestation leads to increased hospital utilization, with longer labor admission-to-delivery times and more extended hospital stays [36]. Multiparous women and those induced for maternal medical indications experience shorter overall time to delivery intervals compared to primiparous women [37]. Additionally, primiparous women are more likely to be induced, particularly for post-maturity [37]. These findings can inform clinical practice, patient counseling, and shared decision-making regarding the induction of labor [37].

### Limitations

This was a retrospective study, and no control over data quality was possible during its design. Therefore, the findings may be subject to selection and information biases. In particular, a selection bias may have influenced the results. We suggest that future prospective studies include control groups managed with observation or oxytocin alone to more clearly determine the comparative effectiveness of Prostin E2 in this clinical setting. Second, the relatively small sample size is a limitation of our study and may impact the statistical power and generalizability of the findings. Future prospective, randomized studies with larger and more balanced cohorts, comprehensive clinical data collection, and structured safety monitoring are warranted to validate and extend these findings.

## 5. Conclusions

In this study, we evaluated the impact of different timings of Prostin E2 administration following Propess insertion in women with unfavorable cervical status undergoing labor induction. The baseline characteristics were generally comparable across groups, except for gestational age, which was higher in the 24 h group. The administration of Prostin E2 at 8 h was associated with a significantly higher Bishop score at 24 h, as well as a shorter induction-to-birth interval, compared to later administration, without increasing the cesarean section rate or compromising neonatal outcomes. Although cesarean section rates tended to be higher with delayed Prostin E2 use, the difference was not statistically significant, and most cesarean deliveries were related to prolonged labor. Neonatal outcomes, including birth weight and Apgar scores, were similar across all groups, suggesting that the timing of Prostin E2 administration does not adversely affect immediate neonatal health. A multivariable regression analysis further identified GA and Bishop score at 24 h as being independent predictors of the induction-to-delivery interval, emphasizing the importance of timely cervical ripening. These findings support the safety and potential clinical benefit of administering Prostin E2 at 8 h post-Propess administration in appropriate patients in order to facilitate a more efficient induction process.

## Figures and Tables

**Table 1:** Baseline characteristics.

	PGE2 (8 h)	PGE2 (12 h)	PGE2 (24 h)	*p*-Value
Case number	19	23	21	
GA (weeks)	38.23 ± 0.34	38.41 ± 0.54	39 ± 0.78	<0.001
Age (years)	30.74 ± 5	28.09 ± 4.23	30.86 ± 6.42	0.153
BMI (kg/m^2^)	29.08 ± 4.1	29.4 ± 5.32	27.6 ± 3.7	0.376
Painless, *n* (%)	18 (94.74)	21 (91.3)	20 (95.24)	0.843
AROM, *n* (%)	14 (73.68)	17 (73.91)	12 (57.14)	0.407
Piton, *n* (%)	13 (68.42)	15 (65.22)	17 (80.95)	0.483

GA: gestational age; BMI: body mass index; AROM: artificial rupture of membranes.

**Table 2:** Maternal outcomes.

Timing of Prostin E2 Addition	8 h	12 h	24 h	*p*-Value
Blood loss (mL)	250.37 ± 110.06	178.26 ± 63.65	261.90 ± 142.21	0.027
Tachysystole, *n* (%)	1 (5.26)	1 (4.35)	1 (4.76)	0.990
Variable deceleration, *n* (%)	3 (15.79)	0 (0)	2 (9.52)	0.160
BS at PGE2 administration (mean ± SD)	3.21 ± 1.47	3.35 ± 2.06	4.67 ± 2.03	0.029
BS after 12 h of Propess use (mean ± SD)	3.84 ± 2.01	3.35 ± 2.06	3 ± 2.57	0.492
BS after 24 h of Propess use (mean ± SD)	9.63 ± 4.22	6.44 ± 2.94	4.67 ± 1.93	<0.001
Mode of birth, *n* (%)				0.464
VED + NSD	17 (89.47)	20 (86.96)	16 (76.19)	
Cesarean section	2 (10.53)	3 (13.04)	5 (23.81)	
Induction-to-birth interval (hours)				
VED + NSD	26.87 ± 9.27	31.89 ± 8.31	44.11 ± 9.18	<0.001
Cesarean section	44.28 ± 5.41	37.84 ± 8.4	47.82 ± 5.26	0.168

BS: Bishop score; VED: vacuum extraction delivery; NSD: normal spontaneous delivery; SD: standard deviation.

**Table 3:** Neonatal outcomes.

Time of Prostin E2 Addition	8 h	12 h	24 h	*p*-Value
Birth weight (g)	2912.58 ± 217.04	3057.22 ± 311.16	3043.48 ± 388.03	0.286
Apgar scores at 1 min (mean ± SD)	7.74 ± 1.05	8 ± 0.74	7.95 ± 1.12	0.659
Apgar scores at 5 min (mean ± SD)	8.89 ± 0.57	8.87 ± 0.46	8.9 ± 0.3	0.964

SD: standard deviation.

**Table 4:** Factors associated with the induction-to-birth interval.

Variable	Univariable			Multivariable		
	Beta	SE	*p*	Beta	SE	*p*
Age	0.38	0.27	0.159	0.34	0.17	0.051
BMI	0.44	0.32	0.173	0.46	0.23	0.053
GA	5.64	2.01	0.007	3.33	1.32	0.015
Blood loss	0.03	0.01	0.006	0.01	0.01	0.169
Painless	3.23	5.86	0.583	3.20	3.85	0.410
AROM	0.11	3.08	0.973	1.73	1.97	0.383
Oxytocin use	11.58	2.80	0.000	2.75	2.15	0.207
Bishop score at Propess	−0.18	0.94	0.849	0.18	0.67	0.792
BS after PGE2 12 h	−1.62	0.62	0.011	0.31	0.51	0.546
BS after PGE2 24 h	−2.22	0.25	<0.001	−1.99	0.28	<0.001

Adjusted for age, BMI, GA, blood loss, painless labor, AROM, oxytocin use, Bishop score at Propess, BS after PGE2 12 h, and BS after PGE2 24 h. GA, gestational age; BMI, body mass index; AROM, artificial rupture of membranes; h, hours.

## Data Availability

All relevant data are reported in the article.

## Abbreviations

The following abbreviations are used in this manuscript:

IOL	induction of labor
PGE2	prostaglandin E2
AROM	artificial rupture of membranes
BMI	body mass index
